# The Value of Models in Orthodontia

**Published:** 1904-10-15

**Authors:** Milton T. Watson

**Affiliations:** Detroit


### THE VALUE OF MODELS IN ORTHODONTIA.

BY MILTON T. WATSON, D. D. S. DETROIT.

Read before the Michigan State Association, June 29th, 1904.

The value of accurate and artistically made models, from an orthodontist's standpoint may, with profit, be considered as follows: First their importance as a means of diagnosis; secondly, their value in aiding one in determining upon a plan of retention after active treatment has been completed; thirdly, as a means of education, both for the profession and the laity, and lastly from a medico-legal aspect.

Taking up the discussion under the first heading, it is, in the light of modern orthodontia, a basic principle that accurate models are essentials to an accurate diagnosis, for, unlike the physician's patients, ours never regain a normal condition when treated in compliance with the demands of an incorrect diagnosis. In cases that are complicated at all, it must be clear then that ultimate success depends in no small way upon a right start, which necessitates the possession of good models and these can be made only from plaster impressions.

The successful retention of a complicated case of mal-occlusion is next to impossible unless a comparative study is made of the changed conditions with the original; and models made from distorted modeling compound impressions do not afford this opportunity. The mischief wrought by the mal-occlusion of the lingual cusp of a single tooth has been sufficient to make disastrous results and real heartaches the experience of many a man in his efforts at retention; and the cause of all this trouble has remained unsolved by many, because the habit of carefully studying real models had not been acquired.

The third division is also one of vast importance, but a five minutes' limit on this paper demands brevity-condensed. Were it not for the careful study of occlusion outside the mouth of the living patients, many of the advances made, not only in orthodontia but in the fields of operative and prosthetic dentistry as well, would not have been so clearly demanded; neither would the evils associated with the extraction of a limited number of teeth have been so clearly understood were it not for the possibilities afforded us by the study of orthodontia models.

The ability to determine accurately what changes have taken place in the position of the teeth, and in the bony tissues surrounding them, and also the ability to determine what changes come about as a result of the natural forces of mastication after all operative procedure has ceased, are possible only where fine models of the original condition are procured, and are alone ample justification for the expenditure of the time and energy necessary to produce such models.

The impression made upon the patient or parents, when they see a model which is really a work of art, is not without value, and I would also ask you to remember that a man who can make a good model, but who does not do it, is quite likely to be a man who will not—even though he can do a really high-class piece of work in restoring the occlusion to normal.

Under certain favorable conditions, work that demands a high degree of skill will command remuneration in proportion, and nothing more surely demonstrates a man to be worthy of the confidence which people must have in him before they are satisfied that he is worthy of his hire, than the possession of a fine collection of beautifully, and with all, and above all, accurately-made models.

In this age when intensity of purpose stands out so conspicuously in all the successful walks of life, nothing short of a high degree of success in the making of models is in harmony with the atmosphere of the modern practice of orthodontia.

The medico-legal consideration presents several points worthy of our attention. Imperfect calcification near the cervical margins is frequently unnoticed by parents until the subsequent delcalcification has progressed far enough to demand attention, and at this point the patient is very liable to attribute the condition to the wearing appliances. But what is to me even more important is the fact that these well-made models reveal the conditions of the gums to no small degree. It is by no means difficult, in many cases where a person masticates only on one side, to tell at a glance which it is, for, on that side the gums are in much firmer contact around the necks of the teeth; while on the other, the soft plaster used for the impression will readily force itself between the gums and teeth for some distance, depending upon how flabby they are, and the condition becomes thus clearly revealed in the model. Gums that are diseased and flabby from other causes than disuse are, of course, equally well shown.

In view of the fact that the legal profession is disgraced by occasional leeches who live, not by the just rewards of honorable and high-class services rendered, but rather by blackmail and bribery to which they resort in order to win malpractice suits, it behooves a man to be ever in possession of accurate models which would be of incalculable value to him were he compelled to face such a suit in which injury to either the teeth or gums was alleged. And, too, if an orthodontic operation has succeeded, unappreciative patients can not avoid the payment of just fees by denying the value of the service rendered.

